# Spatial–Temporal Patterns in the Enteric Pathogen Contamination of Soil in the Public Environments of Low- and Middle-Income Neighborhoods in Nairobi, Kenya

**DOI:** 10.3390/ijerph21101351

**Published:** 2024-10-12

**Authors:** Fanta D. Gutema, Bonphace Okoth, John Agira, Christine S. Amondi, Phylis J. Busienei, Sheillah Simiyu, Blessing Mberu, Daniel Sewell, Kelly K. Baker

**Affiliations:** 1 Department of Occupational and Environmental Health, University of Iowa, Iowa City, IA 52242, USA; kelly-k-baker@uiowa.edu; 2 Department of Microbiology, Immunology and Veterinary Public Health, Addis Ababa University, Bishoftu P.O. Box 34, Ethiopia; 3 African Population and Health Research Center, Nairobi 10787-00100, Kenya; bonphaceo@gmail.com (B.O.); agiradenge@gmail.com (J.A.); amondiogalo632@gmail.com (C.S.A.); pbusienei@aphrc.org (P.J.B.); ssimiyu@aphrc.org (S.S.); bmberu@aphrc.org (B.M.); 4 Department of Biostatistics, University of Iowa, Iowa City, IA 52242, USA; daniel-sewell@uiowa.edu

**Keywords:** detection rate, diversity, enteric pathogens, soil, TaqMan array card, hygienic infrastructure, sanitation

## Abstract

Public spaces in countries with limited societal development can be contaminated with feces containing pathogenic microbes from animals and people. Data on contamination levels, spatial distribution, and the diversity of enteric pathogens in the public settings of low- and middle-income neighborhoods are crucial for devising strategies that minimize the enteric infection burden. The objective of this study was to compare spatial–temporal differences in the detection rate and diversity of enteric pathogens in the public spaces of low- and middle-income neighborhoods of Nairobi, Kenya. TaqMan array card (TAC) molecular assays were employed to analyze soil samples for 19 enteropathogens, along with a selective bacterial culture for pathogenic Enterobacteriaceae. An observational assessment was conducted during every site visit to document the hygienic infrastructure and sanitation conditions at the sites. We detected at least one pathogen in 79% (127/160) and ≥2 pathogens in 67.5% (108/160) of the soil samples tested. The four most frequently detected pathogens were EAEC (67.5%), ETEC (59%), EPEC (57.5%), and STEC (31%). The detection rate (91% vs. 66%) and mean number of enteric pathogens (5 vs. 4.7) were higher in low-income Kibera than in middle-income Jericho. The more extensive spatial distribution of pathogens in Kibera resulted in increases in the detection of different enteric pathogens from within-site (area < 50 m^2^) and across-site (across-neighborhood) movements compared to Jericho. The pathogen detection rates fluctuated seasonally in Jericho but remained at sustained high levels in Kibera. While better neighborhood conditions were linked with lower pathogen detection rates, pathogenic *E. coli* remained prevalent in the public environment across both neighborhoods. Future studies should focus on identifying how the sources of pathogen contamination are modified by improved environmental sanitation and hygiene and the role of these contaminated public environments in enteric infections in children.

## 1. Introduction

Between 1990 and 2019, enteric infections from bacteria, viruses, protozoa, and intestinal helminths caused a total of 6595 million incidences, resulting in approximately 1748 thousand deaths [1]. Most deaths occurred in low- and middle-income countries. Enteric infections affect the regular functions of the intestinal tract and can result in several gastrointestinal manifestations, such as nausea, vomiting, and diarrhea [2]. Enteric pathogens are transmitted from human and animal sources to children through multiple environmental pathways and environmental reservoirs [3,4]. Soil and water, in particular, can be an ecological reservoir for a wide range of pathogenic organisms and play a significant role in mediating the transmission of enteric infections from various sources to humans [5].

Urban and peri-urban public environments shared by many residents are highly susceptible to contamination, even in developed countries where there are no strict environmental hygiene regulations and monitoring plans in place. The soil and water in public environments can become contaminated by animal and human feces when inadequate sanitation access leads to the practice of open defecation and where animals are allowed to roam freely [6,7]. Other factors that may serve as mediators and contribute to the contamination of public and domestic environments with enteric pathogens include the direct release of untreated human feces or wastewater into the environment [8], unsanitary latrine pit emptying [9], latrine flooding [10], and contamination sources in healthcare settings from medical devices and staff, including hospitalized patients [11]. While societal development improves environmental hygiene, it also introduces challenges such as increased urbanization and crowding that can influence the dynamics of enteric pathogen transmission [12,13]. Studies in low-income residential environments in Kenya contaminated by human and animal feces have described a very diverse portfolio of enteric pathogens in soil and surface water, suggesting a potential pathway for the transmission of enteric infections [5,14]. Enteric pathogens have different survival times in the environment with some enteropathogens persisting in the environment for long periods of time [15,16]. Pathogens persisting in the environment can subsequently be transported into drinking water, soil, and food by fomites (such as containers and flood water) and vectors (such as hands and flies) and can ultimately be ingested by humans [13]. Children are disproportionately at risk of exposure to enteropathogens from the soil and water in these residential areas [13,17].

Improvements in household sanitation infrastructure can reduce the burden of enteric infections, although randomized trials report mixed evidence of impact [18,19,20,21]. Critical levels of community-wide sanitation coverage may be required to sufficiently protect the environment and prevent child infections. Less is known about how animal management influences community-level contamination. Investigating the role of societal development (improved infrastructure, human and animal sanitation conditions, and hygiene practices) on the prevalence and distribution of enteropathogens in public environments can improve knowledge about how to reduce children’s exposure to soil- and water-transmitted enteric infections. This study aimed to evaluate the spatial–temporal differences in the detection rate and taxonomic diversity of enteric pathogens between and within low- and middle-income neighborhoods of Nairobi, Kenya. The findings derived from this study provide evidence regarding the magnitude and spatial distribution of enteric pathogens, including in under-researched middle-income neighborhoods, as well as the extent to which moderate improvements in overall societal development influence enteric pathogen prevalence in public environments. This information is crucial for devising tailored interventional measures to mitigate the morbidity and mortality associated with enteric infections in children.

## 2. Materials and Methods

### 2.1. Public Domain Site Identification

**Figure 1:**
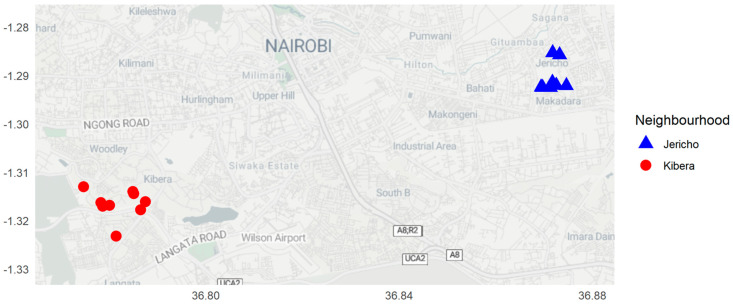
A map showing selected public domain sites in the Kibera and Jericho neighborhoods in Nairobi County, Kenya.

This study is part of a large cohort study, “Statistical and agent-based modeling of complex microbial systems: A means for understanding enteric disease transmission among children in urban neighborhoods of Kenya (PATHOME)”, aimed at investigating and modeling the impact of societal development on enteric pathogen transmission among infants, animals, and the environment. The detailed rationale, study design, eligibility, recruitment, enrollment, and methodological approaches are described in the PATHOME protocol [22]. This specific study used data collected between 14 December 2021 and 28 November 2022 in two neighborhoods, namely, Kibera (a low-income neighborhood) and Jericho (a middle-income neighborhood), located approximately 10 km apart in Nairobi, Kenya. The neighborhoods exhibit distinct differences in terms of socioeconomic advancement both at the domestic household and community levels. Households located in Jericho are constructed with better housing materials and have high access to on-plot toilets and water, and the neighborhoods have wider, more developed roads with formal stormwater drainage systems. In contrast, Kibera is an informal urban settlement characterized by poor-quality housing, inadequate sanitation, insufficient garbage disposal, a lack of formal sewers, and overcrowding (~87,000 people/km) [23]. These differences could result in different levels of the contamination of the public environment with enteric pathogens. Environmental soil samples were collected from 20 public domain sites (10 per neighborhood), which were identified by field enumerators as locations where one or more infants enrolled in this study in the previous month had spent time (noted during a structured observation of households) and had therefore been exposed to soil, surfaces, or water. These sites were typically areas that were very close to the enrolled households, shared by adjacent neighborhoods, and potentially served as children’s playgrounds. A different site was selected in each neighborhood every four weeks, except for the December holidays, for ten consecutive months (Figure 1).

### 2.2. Hygiene Infrastructure and Sanitation Conditions

The designated specific sampling locations were assessed using structured observational tools to characterize the hygiene and sanitation conditions by recording the presence and types of urban infrastructure and the presence of human (including diapers and plastic bags containing feces and individual piles) and domestic animal feces. The urban infrastructure that was evaluated encompassed the presence of residential houses and/or small business enterprises, waste disposal facilities (e.g., garbage dumps), public or communal latrines, shared latrines, large animal confinement/slaughter businesses, small to large wastewater drains, and community recreation and/or use areas.

### 2.3. Sample Collection

Eight soil samples were collected from each identified public site by conducting a transect walk around the approximate 10-m squared area to identify distinct areas. Then, a sterile scoop was used to gently break up the top surface of the ground soil before being inserted into the ground at a 45° to a depth of 5 cm to transfer soil into separately labeled 250 mL Whirl-Pak bags. An attempt was made to collect the same amount of soil per scoop from different locations to obtain an adequate sample of about 30 g of composite sample per site. All the samples were kept in an icebox containing ice packs and transported in a cold chain to the African Population and Health Research Center (APHRC) for the bacteriological analysis within 6 h of collection.

### 2.4. Microbiological Analysis

#### 2.4.1. Sample Preparation

The soil samples were pre-enriched with a non-selective buffered peptone water (BPW) (OXOID, Hampshire, UK) before use to promote the recovery of live, potentially non-replicating bacteria. For each soil sample, three dilutions were made by measuring 25, 2.5, and 0.25 g of soil samples into 225, 250, and 250 mL of BPW to yield 1:10, 1:100, and 1:1000 g of soil to mL of buffer dilutions. All the three dilutions were incubated at 37 °C for 24 h. After the incubation, a 400 µL aliquot of soil with the highest volume (1:10 dilution) was measured into a DNA/RNA shield lysis collection tube (Zymo Research, Tustin, CA, USA) and vortexed. The specimen was then stored at −20 °C until it was shipped to the University of Iowa for the DNA and RNA extraction and molecular analysis. Prior to shipment, 1% Proteinase K (*v*/*v*) (Zymo Research, Tustin, CA, USA) at 20 mg/mL, 4 µL, was directly added to each sample. Additionally, an equal amount (400 μL) of molecular grade water (one per week of sample processing) was added to the tube as a process control during the transfer of the samples into Zymo tubes, and one negative control per 50 samples was extracted following the same protocol to monitor the extraction process.

Additionally, aliquots of 0.1 mL obtained from each of the three BPW primary enrichment cultures were subsequently transferred into three sterile tubes containing 10 mL of secondary selective enrichment broths specific to *Escherichia coli* (*E. coli*), *Salmonella* spp., and *Shigella* spp. utilizing the recommended standard bacteriological methods [24]. The selective enrichment cultures (Rappaport Vassiliadis broth (RVS) (OXOID, UK) for *Salmonella* spp. and *Shigella* spp.) were incubated at 41.5 °C, while the *E. coli* broth (OXOID, UK) (for *E. coli*) was incubated at 37 °C for 24 h. After incubation, a loopful of culture was streaked in duplicate on selective agar and grown overnight at 37 °C to isolate *Salmonella* spp., *Shigella* spp. (Xylose Lysine Deoxycholate (XLD), Remel, KS, USA), and *E. coli* (MacConkey agar, OXOID Auckland, New Zealand). Five to ten presumptive and distinctive colonies on the selective agar were collected from each plate, subcultured into a Tryptic Soya broth (TSB, OXOID, Hampshire, UK), incubated for 24 h at 37 °C, and then preserved as a bacterial culture of glycerol stock at a 1:2 ratio for future use.

#### 2.4.2. TaqMan Array Card Analysis

A custom microfluidic TaqMan card (TAC) containing primers and probes was used to directly and simultaneously detect the enteric pathogens from the pre-enriched soil samples. The TAC contains primers and probes for the major enteric pathogens, namely, Norovirus spp., Adenovirus 40/41, Sapovirus, Enterovirus, Rotavirus, *Salmonella enterica*, *Shigella* spp., *Campylobacter* spp., *Listeria monocytogenes*, *Clostridium difficile*, *Helicobacter pylori*, enterotoxigenic *Escherichia coli* (ETEC), enteropathogenic *E. coli* (EPEC), enteroaggregative *E. coli* (EAEC), Shiga-like toxin-producing *E. coli* (STEC), *Enterocytozoon bieneusi*, *Giardia* spp., *Cryptosporidium* spp., and *Entamoeba histolytica* [22] (Supplementary Table S1). DNA and RNA from the primary enrichment culture of the 1:10 dilution was extracted using a DNA/RNA Pathogen Miniprep kit (Zymo Research Corp, Tustin, CA, USA). For each sample, 40 μL of the DNA and RNA extract was mixed with 60 μL of a PCR mix (50 μL of TaqPath™ ProAmp™ Master Mix (Thermo Fisher Scientific, Vilnius, Lithuania), 0.6 μL of 50 mg/mL bovine serum albumin, and 9.4 μL of nucleic acid-free water). The TaqMan assays were run on a QuantiStudio 7 Pro Real-Time PCR System (ThermoFisher, Chicago, IL, USA) at standard cycling conditions of 95 °C for 10 min of holding, followed by 40 cycles of 95 °C for 15 s and 60 °C for 1 min. The TaqMan Array card assays were validated using 10-fold serial dilutions (10^0^–10^6^ genes per sample) of the positive bacterial controls from the pathogen collection at the Enteric Pathogen Laboratory of the University of Iowa. We used gBlocks gene fragments (IDT, Coralville, IA, USA) for the rest of the enteric pathogens with pathogen-specific sequence inserts targeted by the primer and probe. The process, extraction, and PCR (one 40 μL of molecular grade water per 100 samples) controls were tested on the TAC card to monitor contamination arising from the laboratory conditions. Standard curves were generated for each gene target and the limits of detection, the lowest concentration of the target that can be detected, were determined based on the cycle threshold (Ct) values, where the positive controls were consistently detected but the negative controls were not. The samples were considered positive when the average Ct values were less or equal to the lower limit of detection for each respective target gene (Supplementary Table S2).

#### 2.4.3. Molecular Confirmation of Presumptive Pathogen Colonies

Isolated colonies of presumptive *Salmonella* spp., *Shigella* spp., and *E*. *coli* strains were confirmed positive through the analysis of bacterial DNA using a quantitative real-time PCR. A portion of each glycerol stock was subcultured on selective agar, and then five to ten representative colonies were transferred into 100 μL of molecular grade water, subjected to a 10-min boiling process, and centrifuged at 15,000 rpm for one minute to precipitate cell debris. The resulting supernatant was transferred to a new sterile labeled Eppendorf tube and stored at −20 °C until examined for pathogen confirmation using the qPCR. The qPCR was conducted by mixing 18 μL of mix (10 μL of master mix, 1 μL of TaqMan assay containing specific primers and probes, and 7 μL of nucleic acid-free water) with 2 μL of the DNA template of each bacterial isolate in wells of a 0.2 mL 96-well PCR plate. The same qRT-PCR primers and probe used in the TAC pre-enrichment qPCR were used to ensure a comparability of detection between the two methods.

### 2.5. Data Management and Analyses

The data were entered into standardized forms using Kobo Toolbox software (version 2.023.48). The data cleaning of the exported datasets was executed using a Microsoft Excel spreadsheet. Prior to the analysis, the observation data from the public domain were thoroughly evaluated for their completeness and consistency by calculating the frequencies of each variable and then combined with the microbiological data for analysis. Data from 20 public sites with complete information were included in the analysis to examine the difference in pathogen diversity and distributions per public site and sample and between the neighborhoods.

The data analysis results were generated using the statistical R software version 4.1.2 (R Foundation for Statistical Computing, Vienna, Austria). Descriptive statistics for the pathogen detection rates at the sample and site levels were computed, analyzing each neighborhood separately. Due to the high levels of sparsity in some pathogens, we computed credible intervals for pathogen prevalence in the soil using the Bayesian hierarchical model. The presence/absence of each pathogen at each sample or site was modeled using a Bernoulli distribution: each of the pathogen-specific probabilities of a positive (detect) came from a beta distribution common to all pathogens. We used an improper uniform prior on the shape parameters of this beta distribution. In this way, we were able to leverage information across pathogens to assist with those that were too sparse to reliably analyze separately. To compare neighborhoods at both the sample and site levels, we computed the estimates, credible intervals, and probability of direction (i.e., the posterior probability that we know the sign of the difference in prevalences) for the difference between neighborhood prevalences. We also compared selective culture detection rates at the site level using this same Bayesian hierarchical model.

Pathogen diversity is a disease-targeted indicator of taxonomic diversity or species richness outcomes used in microbial community characterization studies. We plotted the distribution of pathogen diversity, as measured by the total number of different pathogens detected in a soil sample (possible range from zero to nineteen per qPCR assay design). We then performed Bayesian bootstrapping on diversity at both the sample and site levels. From this, we obtained an estimated credible interval and probability of direction of the difference in diversity between the two neighborhoods. To examine the spatial relationship between the pathogens in soil samples, we performed a Monte Carlo microsimulation study to investigate the increase in diversity that occurs due to local movement, i.e., moving from one sample to another within the same site, and due to non-local movement, i.e., moving from one sample to another in different sites while staying in the same neighborhood. We additionally examined the increase in pathogen diversity by moving from a location in Jericho to a location in Kibera and vice versa.

Finally, we assessed the temporal seasonality distributions of the most frequently detected pathogens by fitting generalized linear models based on the Bernoulli distribution with weakly regularizing priors. For each of the two neighborhoods and each of EAEC, ETEC, EPEC, and STEC, we predicted presence/absence using periodic B-splines on the day of the year (1–365). Leave-one-out cross-validation was used to select the number of knots.

## 3. Results

### 3.1. Hygienic Infrastructure and Sanitation Conditions

Of the twenty public domain sites targeted, complete observational data were obtained for eighteen public sites (nine per study area). Table 1 presents a summary of the observed public infrastructure and sanitation conditions within or near the public domain soil sampling locations. Residences and/or small businesses were observed near all the identified public domain sites in Jericho and at 78% of the sites in Kibera. In Jericho, we observed a higher percentage of open community recreation/use areas (67%). A public latrine was observed at two locations in Jericho and at one site in Kibera. An equal number of trash dumps (n = 4) were observed in both locations. There were visible human and/or animal fecal materials at 44% of the sites in both neighborhoods and at all sites in Kibera. Visible surface water was observed in 56% of the sites in Jericho and in all the sites in Kibera where there was higher (89%) contamination with trash.

### 3.2. Pathogen qRT-PCR Detection Rate from Pre-Enriched Soil

Among the enteric pathogens investigated, the most frequently detected by the TAC analysis in individual pre-enriched soil samples were bacterial pathogens, predominantly EAEC (67.5%) followed by ETEC (59%), EPEC (57.5%), STEC (31%), *Campylobacter* spp. (21%), *Shigella* spp. (5%), and *Salmonella* spp. (3%) (Table 2). In all the soil samples tested, *Sapovirus* spp., *L. monocytogenes*, *Cryptosporidium* spp., and *Clostridium difficile* were not detected. The probability of detection in individual Kibera soil samples compared to Jericho soil samples was higher for EAEC (PD = 0.38), ETEC (PD = 0.35), EPEC (PD = 0.31, *Shigella* spp. (PD = 0.10), and STEC (PD = 0.03) (Table 3). The differences in detection did not vary significantly for the other organisms. The most common pathogens were detected at almost all sites where clusters of individual neighboring (within 10 m) samples were collected in both Jericho and Kibera (Table 2).

### 3.3. Pathogen qRT-PCR Detection Rate from Selective Cultures

The isolation of *E. coli*, *Salmonella* spp., and *Shigella* spp. from soil samples (N = 160), utilizing rigorous subculturing procedures that included secondary enrichment and selective plating, resulted in a higher frequency of detection of the three bacteria species in both study sites (Table 4). The detection rate of colonies matching *Salmonella* spp. (87.5% versus 61.3%) and *Shigella* spp. (35% versus 17.5%) phenotypes was higher in low-income Kibera than in middle-income Jericho. *E. coli* colonies were detected in almost all soil samples at an equal proportion in both sites. In contrast to the TAC testing of the pre-enriched samples, almost all the presumptive *E. coli*, *Shigella* spp., and *Salmonella* spp. colonies tested negative using the qPCR, suggesting the need for further genetic profiling of the presumptive strains. Only three presumptive *Salmonella* spp. isolates were found positive for the *Salmonella enterica* ttr pathogenicity gene. As a result, we utilized the results of the TAC analysis to examine the diversity and temporal and spatial distributions of the pathogens per soil sample and public site and compared them for any differences between the neighborhoods.

### 3.4. Pathogen Diversity

**Figure 2:**
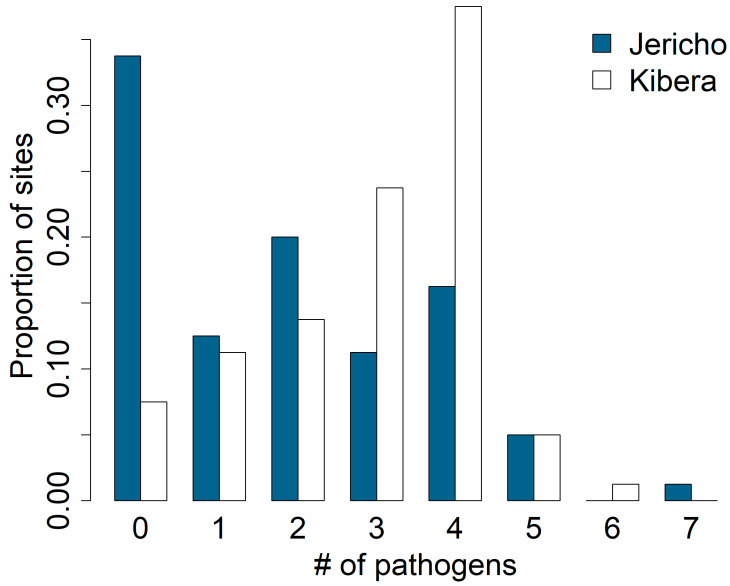
Distribution of the number of pathogens detected across sampling sites in Jericho and Kibera, Nairobi, Kenya (N = 160).

Out of the 160 soil samples examined, 79% (127/160) contained at least one type of pathogen and 67.5% (108/160) contained two or more types of pathogens. The rate of recovering at least one type of enteric pathogen was 91% (73/80) in Kibera and 66% (53/80) in Jericho. The mean pathogen diversity detected within each public domain site (N = 20) was 5 pathogen types in Kibera versus 4.7 types in the Jericho soil (Supplementary Table S3), leading to an estimated difference in the mean number of pathogens of 0.3 (95% CI: −1.1, 1.13), with a 0.611 posterior probability that the mean number of pathogens was higher in Kibera (Figure 2). Since the virus and protozoan detection frequencies were similar between the neighborhoods, this difference reflects measurable differences in bacteria pathogen detections between neighborhoods.

### 3.5. Spatial Distributions

We further evaluated the spatial distribution of the most frequently detected enteric pathogens across the sampling locations and found that they were more widely distributed in Kibera than in Jericho. In both study sites, the four most widely distributed pathogens were EAEC, EPEC, ETEC, and STEC, with a variable number of pathogens being detected per the eight samples collected from each respective public site (ranging from zero to eight). *Campylobacter*, *Shigella* spp., and *Salmonella enterica* were observed to be sparsely distributed. *Shigella* was detected in three sites, namely sites 2, 5 and 9, in Kibera, whereas it was not detected in any of the public sites in Jericho (Table 5).

**Figure 3:**
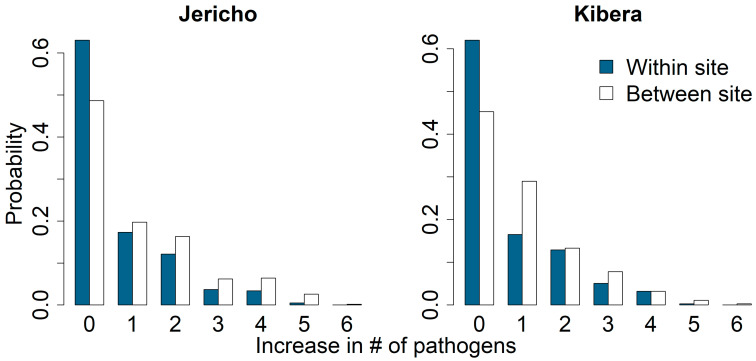
Probability of exposure to one or more new types of enteric pathogens from public soil if someone moves between locations within one residential site (area < 50 m^2^) versus between neighborhood sites in Jericho and Kibera.

**Figure 4:**
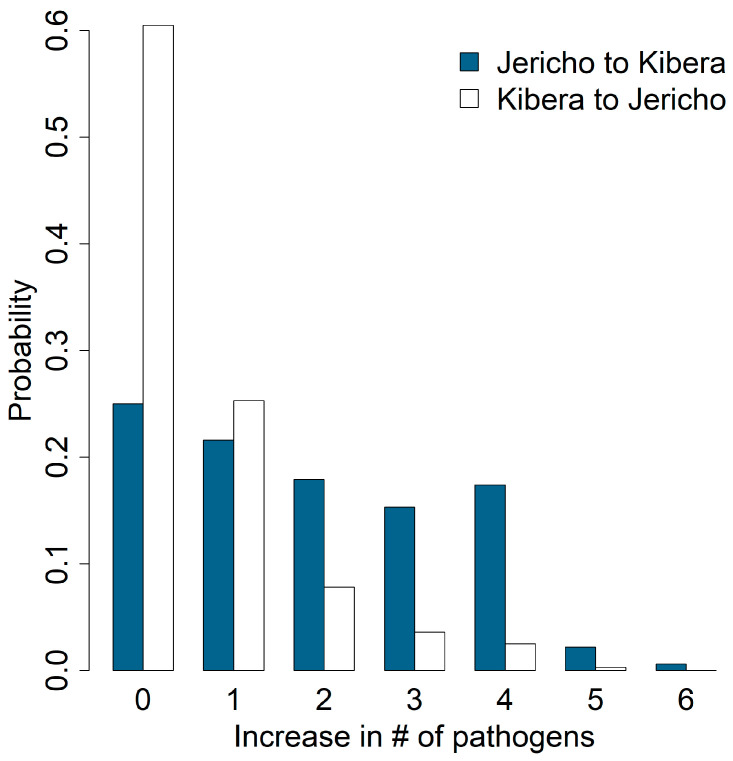
Probability of exposure to one or more new types of enteric pathogens in public soil if someone moves between neighborhoods from Jericho to Kibera versus Kibera to Jericho.

Figure 3 shows the results from the Monte Carlo study on the increase in enteric pathogen diversity due to movements within neighborhoods. Such movements were either constrained to within-site (10 m^2^) or between-site movements. On average, we would expect the diversity of exposure to increase by 0.69 and 0.72 for within-site movements in Jericho and Kibera, respectively, and by 1.11 and 0.99 for between-site movements in Jericho and Kibera, respectively. The reasons for these small increases appear to be contrasting for Jericho and Kibera: for Jericho, one is less likely to experience new pathogens through within- or across-site movements, whereas in Kibera one is more likely to already have been exposed to multiple pathogens in the origin location. This is further evidenced in Figure 4, which shows the distribution of the increase in pathogen diversity when moving from a sample in one neighborhood to a different sample in the other neighborhood. On average, moving from Jericho to Kibera led to an increase in diversity of 1.88, while from Kibera to Jericho only led to an average increase of 0.63, less than a local move within Kibera. This further highlights the clear differences in pathogen prevalences in Kibera compared to Jericho.

### 3.6. Temporal Distributions of Major Enteric Bacteria

**Figure 5:**
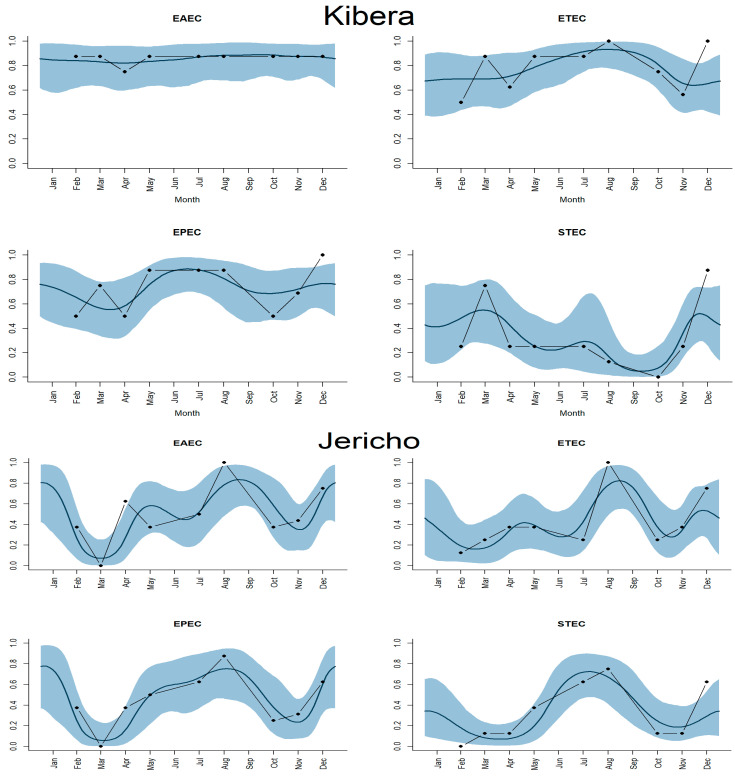
Seasonal trends in the detection of EAEC, ETEC, EPEC, and STEC in public soil in Jericho and Kibera, Nairobi, Kenya. EAEC = enteroaggregative *E. coli*; ETEC = enterotoxigenic *E. coli*; EPEC = enteropathogenic *E. coli*; and STEC = Shiga toxin-producing *E. coli*.

Figure 5 shows the seasonality trend for EAEC, ETEC, EPEC, and STEC. Seasonal patterns are apparent for Jericho, but not for Kibera. While the Kibera prevalence rates tend to stay high year-round, there appear to be dips around March and November with a peak around August and September for Jericho.

## 4. Discussion

We investigated the spatial–temporal differences in the detection rate, species diversity, and contamination level of enteric pathogens in the soil between and within public domain sites in low- and middle-income neighborhoods of Nairobi, Kenya. The enteric pathogens examined in this study are known to cause enteric infections in children in most low-income and middle-income countries [25,26,27,28,29,30,31] and in some developed countries where sanitation infrastructure is limited or lacking [32,33]. Collectively, our pathogen detection rates from public domain sites suggest that soil could serve as an important mode of transmission for enteric infections in children and that the risks for children in middle-income communities with more developed sanitary infrastructure are lower. Our study revealed considerable differences between the two neighborhoods, with a higher detection rate, species diversity, and spatial distribution of enteric pathogens in the low-income informal settlement of Kibera than middle-income Jericho. We also found seasonal differences in pathogen contamination between the neighborhoods.

Several studies have investigated the prevalence of fecal contamination in public domain soil, primarily utilizing fecal indicator organisms like *E. coli* as an indicator of contamination. All studies in low-income communities have found the fecal indicator *E.coli* in all soil samples collected from children play spaces and concluded they reflect environmental contamination and the need for improved sanitation and hygiene practices [34]. Although the examination of fecal indicator bacteria, such as *E. coli*, in soil can yield valuable insights into the contamination levels in some circumstances, these bacteria can be naturalized microbiota of the ecosystem, and indicator assays cannot attribute their presence to human, animal, or environmental origin [35,36]. In this study, we found no difference in general *E. coli* in Kibera and Jericho, even though Jericho has comprehensive household sanitation coverage and low open defecation, indicating *E. coli* was not useful as an indicator of human feces contamination. Additional limitations included inaccuracy in predicting the presence of specific enteric pathogens. This lack of specificity can result in the overestimation or underestimation of the health risks associated with fecal contamination in soil. Assessing for specific enteric pathogens provided a more comprehensive understanding of soil contamination with the relevant pathogens and their potential impact on public health [37].

Our enrichment plus PCR-based detection approach identified at least one enteric pathogen in 79% of the public domain soils in this study, which was low compared to 96% (N = 28) detection rates in the soil collected from compounds in Kibera in 2020 [14] and 91% (N = 179) rates in the soil collected from shared latrine entrances and household compounds in Maputo, Mozambique [6]. However, the pathogen detection rates in Kibera specifically (91%) were comparable. The overall rates, including the 66% in Jericho, were higher than the 32.5% prevalence of enteric pathogens in soil in India [38] and the 33% in public soil in Kisumu, Kenya [5]. The difference in the detection rates between the studies could be attributed to differences in sample size, detection methods, targeted organisms (indicator versus pathogenic organisms), and whether the contamination level surpassed the methodological limit of detection.

In both of our study neighborhoods, EAEC, ETEC, EPEC, and STEC (hereafter referred as “pathogenic *E. coli*”) were widely distributed, but they were more consistently detected within residential child play spaces in Kibera than Jericho. Consistent with our findings, previous studies have reported a high prevalence of pathogenic *E. coli* compared to other enteric pathogens in the public environment [38]. This could be due to the long survival time of pathogenic *E. coli* in the environment [39]. Our use of pre-enrichment to improve the recovery of viable bacteria in the soil could also have biased detection frequencies in favor of bacteria since they can replicate to high detectable concentrations under enrichment conditions, but viruses and protozoans cannot. We may have underestimated the virus and protozoan contamination frequencies. While we may have missed some important spatial patterns for viruses and protozoans, it would not alter our conclusions about the differences in the spatial distribution of enteric bacteria within and across neighborhoods. The detection of *Shigella* spp. in Kibera only is particularly interesting, because *Shigella* spp. would have benefitted from the enrichment step and, if present, could also have resulted in high detection frequencies across neighborhoods. *Shigella* spp. are considered to be a human-specific bacteria, unlike most pathogenic *E. coli*, suggesting that they are a more discriminatory indicator of human fecal contamination. The non-detection of *Shigella* spp. in Jericho may reflect reductions in the environmental hazards generated by improvements in household sanitation access.

While pathogenic *E. coli* and *Shigella* spp. were the most prevalent pathogens, a diverse range of enteric pathogens were also present in the soil, with higher taxonomic pathogen diversity in the Kibera soil than in the Jericho soil. Finding a co-occurrence of pathogens is in agreement with our previous study in Kisumu, Kenya [5], and studies conducted in Bangladesh [40], India [38], and Myanmar [41], which reported the occurrence of multiple enteric pathogens in public areas and children’s playgrounds. The occurrence and spread of human and animal feces containing enteric pathogens in commonly shared public environments [38] increases the probability that children who play near these sites will be exposed to multiple pathogens, leading to infection or co-infection [17,42]. A recent study conducted in Bangladesh demonstrated that co-infection patterns for different pathogen-to-pathogen pairs, such as ETEC and EPEC, are common in children and are associated with diarrhea [43]. As acknowledged above, the underdetection of viruses and protozoans could have resulted in the underestimation of the pathogen diversity in the soil. However, the similar detection levels in each neighborhood suggest any effect on the spatial analysis results would have been a bias towards non-difference rather than overstating neighborhood differences. Studies examining the relationships between the diversity in enteric pathogens in child play spaces and subsequent enteric infections in children, like the PATHOME study [22], are needed to test the hypothesis that neighborhood contamination is an important cause of child infections.

Our evidence on pathogens in neighborhoods with comprehensive infrastructure coverage adds unique evidence for testing counterfactual hypotheses about the capacity of neighborhood development to lower enteric pathogen species diversity in the environment and ergo exposure and infections in children. Several studies have found that higher thresholds of neighborhood latrine coverage, for example, lead to reductions in diarrhea [44]. However, the comprehensive overhaul of housing and infrastructure across large neighborhoods is a daunting and costly task, especially in informal urban slums. A practical question then for implementers is how large a spatial area must receive development to catalyze changes in environmental safety and health. One of our prior studies in Kenya found that contact with the soil from multiple public sites in a neighborhood increased the probability of exposure to new pathogen species in soil, although sampling sites were separated by distances of 100 m or more [42]. This new study examined the between-neighborhood, within-neighborhood (area 1–2 km^2^), and within-site (area < 50 m^2^) spatial distributions of enteric pathogens and pathogen diversity. The most widespread pathogens, EAEC, EPEC, ETEC, and STEC, were ubiquitous in each neighborhood, but had a wider distribution within Kibera. For instance, in Kibera, EAEC was detected in more than 86% of all samples with similar detection rates in each site (with a range of six to eight positives/site). In Jericho, the ~50% EAEC detection came from only nine sites with a wide range of within-site detection (two to eight positives/site). EPEC and ETEC had similar spatial patterns, while *Campylobacter* and *Shigella* spp. occurred in more sparse and spatially clustered patterns.

These pathogen-specific spatial patterns led to differences in the probability of increased pathogen diversity. In brief, a child in Jericho must move around a large area of Jericho, or go to Kibera, to be exposed to an equal number of enteric pathogen types (for example, EAEC, Norovirus, and *Shigella*) as a child in Kibera moving within one public play site near their home. However, children in both neighborhoods only develop an enteric infection if they ingest a sufficient volume of soil containing an infectious concentration of viable pathogens.

The pathogen detection rates in Kibera tended to stay high year-round. However, a seasonal trend was observed for pathogenic *E. coli* detection in Jericho, with dips around the dry months of February to March and October to November and a peak during the wetter months of May to September. Pathogenic *E. coli* strains are capable of surviving for extended periods outside the intestinal tract and reproducing in soil under diverse ecological conditions, including tropical, subtropical, and temperate climates. However, several ecological factors, such as temperature, soil moisture, and rainfall, can influence the growth and survival of pathogenic *E. coli* in the environment, resulting in variable distributions of the pathogens across the year [45]. In a similar manner, the frequency of *E. coli* infections may exhibit seasonal variation. One systematic review indicated that the incidence of diarrheagenic *E. coli* increases by 8% for a 1 °C increase in mean monthly temperature, suggesting environmental temperature enhances pathogen survival and growth [46]. Other studies have proposed a “concentration-dilution hypothesis” that states that pathogens build up in the environment during dry seasons and are at their highest concentration in water when heavy rainfall flushes pathogens across the environment. As rain continues, the concentration of pathogens in the environment becomes diluted, resulting in a lower incidence rate during the rainy season and a higher incidence rate during the dry or post-dry flood season [47]. One study conducted in Kenya observed the highest incidence of infection among children with pathogenic *E. coli* during the dry season [48].

Our results disagree with these theories in several ways. First, we did not observe fluctuations in the pathogens in the environment in Kibera where open human and animal waste disposal is common. If we assume that pathogens are introduced into the environment through open human and animal fecal disposal daily in this setting, then our data suggest a net sum balance where pathogens are deposited at a similar rate with which they are decaying or being washed away. The implications for child exposure and infection would be high levels of non-seasonal enteric pathogen transmission, which is supported by PATHOME and other community-based epidemiological studies of infection carriage. Second, in Jericho, where seasonal patterns did occur, we observed the sustained detection of pathogenic *E. coli* in the environment between roughly 4 and 8 weeks after the onset of the rainy season through four months after, and again in November to December during what is considered the “short rains” season. This observation does align with microbiological knowledge that water enables bacterial replication and viability and this biological influence of climate conditions may minimize a dilution effect from sustained rainfall. Third, we did not observe a buildup of pathogens in the dry season, although this may be due to a lower rate of human feces replacement in the environment in Jericho. This was substantiated during our observational assessment that no human feces were visibly observed in Jericho, indicating a difference in access to sanitation services. While neighborhood differences and seasons were quite distinct, our sampling size was limited to one representative site per neighborhood each month and eight soil samples across that site. Our observations could be biased if this one sampling location was not representative of other neighborhood conditions. Further studies are needed to determine the determinants and seasonal pattern of occurrence of the pathogens.

## 5. Conclusions

The observed differences in the detection rate, diversity, and spatial distributions of the enteric pathogens in the public environment between the Kibera and Jericho neighborhoods substantiate the hypothesis that societal development substantially decreases environmental contamination with human and animal feces. Due to the limited number of public domain sites (10 per neighborhood), we did not estimate the associations between sanitation infrastructure and hygiene conditions with pathogen presence. However, the differences could be attributable to factors such as the inadequate sanitation, inadequate waste management, and flooding in Kibera [23,49]. While improved waste and wastewater infrastructure likely contributed to lower pathogen detection rates in Jericho, the widespread and sustained occurrence of pathogens like pathogenic *E. coli* indicates that conditions are still not sufficient for eliminating exposure to enteric pathogens. Other feces sources, such as domestic animals, still sustain health risks. The widespread occurrence of multiple pathogens in both low- and middle-income public environments may pose a risk of enteric infections to children who play in these areas, with the risk being lower for middle-income children at seasonal times of the year. Future studies should focus on quantifying the role of the contaminated public environment exposure pathways mediated by soil and surface water and the associated risks. This will be essential to inform tailored intervention strategies for preventing enteric infections.

## Figures and Tables

**Table 1:** Observed public hygiene infrastructure and sanitation conditions in 18 public domain study sites in Jericho and Kibera, Nairobi.

Variables	Jericho (n = 9)	Kibera (n= 9)	Total (N = 18)
	Number (%)	Number (%)	Number (%)
Residences and/or small businesses	9 (100)	7 (78)	16 (89)
Trash dumps	4 (44)	4 (44)	8 (44)
Public/communal latrines	2 (22)	1 (22)	3 (17)
Large operation animal confinement	0 (0)	0 (0)	0 (0)
Major wastewater drains	2 (22)	4 (44)	6 (33)
Community recreation or use area	6 (67)	1 (22)	7 (39)
Presence of feces	4 (44)	4 (44)	4 (44)
Human feces	0 (0)	3 (33)	3 (17)
Chicken feces	1 (11)	2 (22)	3 (17)
Goat	0 (0)	0 (0)	0 (0)
Cow	2 (22)	0 (0)	0 (0)
Dog	2 (22)	4 (44)	6 (33)
Cat	0 (0)	1 (11)	1 (6)
Other feces	0 (0)	1 (11)	1 (6)
Presence of surface water	5 (56)	9 (100)	14 (78)
Standing water area	1 (11)	2 (22)	3 (17)
Open drain water	4 (44)	8 (89)	12 (67)
Covered drain water	0 (0)	0 (0)	0 (0)
Sewage canal water	0 (0)	4 (44)	4 (22)
Natural streams	0 (0)	2 (22)	2 (11)
Pond	0 (0)	0 (0)	0 (0)
Surface water with trash	4 (44)	8 (89)	12 (67)

**Table 2:** Detection rate of enteric pathogens in 160 public domain soil samples from 20 sites in Jericho and Kibera, Nairobi, Kenya.

Pathogen	Jericho				Kibera			
	Site (n = 10)		Sample (n = 80)		Site (n = 10)		Sample (n = 80)	
	Positive	Rate ^†^ (95% CI)	Positive	Rate (95% CI)	Positive	Rate (95% CI)	Positive	Rate (95% CI)
EAEC	9	82 (55, 98)	39	47 (37, 58)	10	95 (76, 100)	69	85 (77, 92)
ETEC	10	91 (67, 100)	33	41 (31, 52)	10	95 (76, 100)	61	75 (65, 84)
EPEC	9	82 (55, 98)	34	40 (30, 51)	10	95 (76, 100)	58	72 (61, 81)
STEC	8	73 (45, 94)	24	29 (20, 39)	9	86 (60, 99)	26	32 (22, 43)
*Campylobacter* spp.	2	21 (4, 27)	8	9.9 (5, 17)	1	11 (1, 35)	6	8 (3, 14)
*Shigella* spp.	0	3 (0, 19)	0	0 (0, 2)	3	30 (8, 59)	8	10 (5, 17)
*Salmonella enterica*	2	21 (4, 27)	3	4 (0, 9)	2	21 (3, 48)	2	3 (0.4, 7)
*Enterocytozoon bieneusi*	2	21 (4, 27)	2	3 (0.4, 7)	0	2 (0, 15)	0	0 (0, 2)
*Entamoeba histolytica*	1	12 (1, 35)	1	1.6 (0, 5)	1	11 (1, 35)	1	2 (0, 5)
Enterovirus	0	3 (0, 19)	0	0 (0, 2)	2	21 (3, 48)	2	3 (0.4, 7)
*Giardia* spp.	1	12 (1, 35)	1	2 (0, 5)	0	2 (0, 15)	0	0 (0, 2)
*Rotavirus* spp.	1	12 (1, 35)	1	2 (0, 5)	0	2 (0, 15)	0	0 (0, 2)
*Helicobacter pylori*	1	12 (1, 35)	1	2 (0, 5)	0	2 (0, 15)	0	0 (0, 2)
Adenovirus 40/41	0	3 (0, 19)	0	0 (0, 2)	1	11 (1, 35)	1	2 (0, 5)
Norovirus GI/GII	1	12 (1, 35)	1	2 (0, 5)	0	2 (0, 15)	0	0 (0, 2)
*Clostridium difficile*	0	3 (0, 19)	0	0 (0, 2)	0	2 (0, 15)	0	0 (0, 2)
*Cryptosporidium* spp.	0	3 (0, 19)	0	0 (0, 2)	0	2 (0, 15)	0	0 (0, 2))
*Listeria monocytogenes*	0	3 (0, 19)	0	0 (0, 2)	0	2 (0, 15)	0	0 (0, 2)
Sapovirus	0	3 (0, 19)	0	0 (0, 2)	0	2 (0, 15)	0	0 (0, 2)

CI = credible interval; EAEC = enteroaggregative *E. coli*; ETEC = enterotoxigenic *E. coli*; EPEC = enteropathogenic *E. coli*; and STEC = Shiga toxin-producing *E. coli*. ^†^ Rates provided are those estimated through the hierarchical Bayesian model rather than empirical rates.

**Table 3:** Estimated difference in the probability of detection of enteric pathogens in 160 public domain soil samples in Kibera and Jericho, Nairobi, Kenya.

Pathogen	Probability Detection		Probability Difference (PD) (95% CI)
	Kibera (n = 80)	Jericho (n = 80)	
EAEC	0.85	0.47	0.38 (0.24, 0.51)
ETEC	0.75	0.40	0.35 (0.21, 0.49)
EPEC	0.72	0.41	0.31 (0.16, 0.45)
*Shigella* spp.	0.10	0.00	0.10 (0.04, 0.17)
STEC	0.32	0.29	0.03 (−0.11, 0.17)
Enterovirus	0.02	0.00	0.02 (−0.01, 0.07)
Adenovirus 40/41	0.01	0.00	0.01 (−0.01, 0.05)
*Entamoeba histolytica*	0.01	0.01	0.00 (−0.04, 0.04)
*Clostridium difficile*	0.00	0.00	0.00 (−0.02, 0.02)
*Listeria monocytogenes*	0.00	0.00	0.00 (−0. 02, 0.02)
Sapovirus	0.00	0.00	0.00 (−0.02, 0.02)
*Cryptosporidium* spp.	0.00	0.00	0.00 (−0.02, 0.02)
*Salmonella enterica*	0.03	0.04	−0.01 (−0.07, 0.04)
*Helicobacter pylori*	0.00	0.01	−0.01 (−0.05, 0.01)
Rotavirus	0.00	0.01	−0.01 (−0.05, 0.01)
Norovirus GI/GII	0.00	0.01	−0.01 (−0.05, 0.01)
*Giardia*	0.00	0.01	−0.01 (−0.05, 0.01)
*Campylobacter* spp.	0.07	0.09	−0.02 (−0.11, 0.06)
*Enterocytozoon bieneusi*	0.00	0.03	−0.03 (−0.07, 0)

EAEC = enteroaggregative *E. coli*; ETEC = enterotoxigenic *E. coli*; EPEC = enteropathogenic *E. coli*; and STEC = shiga toxin-producing *E. coli.*

**Table 4:** Detection rate of *E. coli*, *Salmonella*, and *Shigella* phenotypes in public domain soil following secondary enrichment and selective plating in Jericho and Kibera, Nairobi, Kenya (N = 160).

Pathogen Phenotype	Jericho (n = 80)		Kibera (n = 80)		Kibera–Jericho
	Positive	Rate ^†^ (95% CI)	Positive	Rate (95% CI)	Rate Difference (95% CI)
*Salmonella*	49	61.5 (51, 72)	70	87 (79, 93)	25 (13, 38)
*Shigella*	14	19.1 (11, 29)	28	38 (27, 49)	19 (5, 33)
*E. coli*	80	99 (95, 100)	79	97 (92,100)	−1 (−7, 3)

CI = credible interval. ^†^ Rates provided are those estimated through the hierarchical Bayesian model rather than empirical rates.

**Table 5:** Frequency of detection of major enteric pathogens from soil samples within and across public domain sites in Jericho and Kibera, Nairobi, Kenya (N = 160).

Neighborhood	Site	Number of Pathogens per Eight Samples Collected at Each Public Site						
		EAEC	EPEC	ETEC	STEC	*Campylobacter*	*Shigella*	*Salmonella*
**Jericho** **(middle-income)**	Site 1	*6*	**5**	**6**	**5**	**0**	**0**	***2***
	Site 2	*3*	3	1	0	0	0	0
	Site 3	0	0	2	1	4	0	0
	Site 4	*5*	3	3	1	4	0	0
	Site 5	*3*	4	3	3	0	0	0
	Site 6	*4*	5	2	5	0	0	0
	Site 7	*8*	7	8	6	0	0	1
	Site 8	*3*	2	2	1	0	0	0
	Site 9	*5*	4	5	2	0	0	0
	Site 10	*2*	1	1	0	0	0	0
	**Total**	**39**	**34**	**33**	**24**	**8**	**0**	**3**
**Kibera** **(low-income)**	Site 1	*7*	8	8	7	0	0	0
	Site 2	*7*	4	4	2	0	1	0
	Site 3	*7*	6	7	6	0	0	0
	Site 4	*6*	4	5	2	6	0	0
	Site 5	*7*	7	7	2	0	6	1
	Site 6	*7*	7	7	2	0	0	0
	Site 7	*7*	7	8	1	0	0	1
	Site 8	*7*	4	6	0	0	0	0
	Site 9	*8*	7	7	3	0	1	0
	Site 10	*6*	4	2	1	0	0	0
	**Total**	**69**	**58**	**61**	**26**	**6**	**8**	**2**

The grey color represents the sites contaminated with at least with one enteric pathogen, while the white color represents sites where no pathogen was detected in this study. The number in each cell represents the number of soil samples at public domain sites that were positive for each respective pathogen.

## Data Availability

The authors confirm that the necessary information to replicate the outcomes of this study is available in the manuscript.

## Supplementary Materials

The following supporting information can be downloaded at https://www.mdpi.com/article/10.3390/ijerph21101351/s1, Table S1: Primers and probes used to test enteric pathogens in soil samples; Table S2: The limit of detection and the average Ct-value of the enteric pathogens for the positive samples; Table S3: Spatial distribution and diversity of enteric pathogens in public domain sites soil in Kibera and Jericho, Kenya (N = 160).
