# Supplementary material for: Spatial–Temporal Patterns in the Enteric Pathogen Contamination of Soil in the Public Environments of Low- and Middle-Income Neighborhoods in Nairobi, Kenya
Source: Int J Environ Res Public Health. 2024 Oct 12;21(10):1351. doi: 10.3390/ijerph21101351 (PMC11506941; doi:10.3390/ijerph21101351)
Supplement: Supplementary file 1 [file ijerph-21-01351-s001.zip › Supplementary file 1.pdf]

Pathogens	Gene (s)	Forward Primer1/2	Reverse Primer 1/2	Probe Sequence 1/2
Adenovirus 40/41	*Fiber Gene*	AACTTTCTCTCTTAATAGACGCC	AGGGGGCTAGAAAACAAAA	CTGACACGGGCACTCT
Sapovirus	*RdRp*	GAYCASGCTCTCGCYACCTA	CCCTCCATYTCAAACACTA	CCRCCTATRAACCA
		TTGGCCCTCGCCACCTAC		
Shigella/EIEC backbone	*ipaH*	CCTTTTCCGCGTTCCTTGA	CGGAATCCGGAGGTATTGC	CGCCTTTCCGATACCGTCTCTGC
Shigella/EIEC plasmid	*virG*	TCAGAAAGGTAATTGGCATGGA	AGAACCGCGCCCAAAGA	AGGGCGGAATATT
Norovirus GI	*ORF 1-2*	CGYTGGATGCGNTTYCATGA	CTTAGACGCCATCATCATTYAC	TGGACAGGAGATCGC
Norovirus GII	*ORF 1-2*	CARGARBCNATGTTYAGRTGGATGAG	TCGACGCCATCTTCATTCACA	TGGGAGGGCGATCGCAATCT
Rotavirus	*NSP3*	ACCATCTWCACRTRACCCTCTATGAG	GGTCACATAACGCCCCTATAGC	AGTTAAAAGCTAACACTGTCAAA
Cryptosporidium spp.	*18s rRNA*	GGGTTGTATTTATTAGATAAAGAACCA	AGGCCAATACCCTACCGTCT	TGACATATCATTCAAGTTTCTGAC
Enterocytozoon bieneusi	*ITS*	TGTGTAGGCGTGAGAGTGTATCTG	CATCCAACCATCACGTACCAATC	CACTGCACCCACATCCCTCACCCTT
Entamoeba histolytica	*18S rRNA*	ATTGTCGTGGCATCCTAACTC	GCGGACGGCTCATTATAACA	TCATTGAATGAATTGGCCATTT
EAEC	*aaic/aatA*	ATTGTCCTCAGGCATTTCAC	ACGACACCCCTGATAAACAA	TAGTGCATACTCATCATTTAAG
		CTGGCGAAAGACTGTATCAT	TTTTGCTTCATAAGCCGATAGA	TGGTTCTCATCTATTACAGACAGC
Enterovirus	*5'UTR*	CCCTGAATGCGGCTAATCC	GCGATTGTCACCATWAGCAG	CCGACTACTTTGGGWGTCCGT
EPEC	*eae*	CATTGATCAGGATTTTTCTGGTGATA	CTCATGCGGAAATAGCCGTTA	ATACTGGCGAGACTATTTCAA
EPEC	*bfpa*	TGGTGCTTGCGCTTGCT	CGTTGCGCTCATTACTTCTG	CAGTCTGCGTCTGATTCCAA
ETEC	*LT*	TTCCCACCGGATCACCAA	CAACCTTGTGGTGCATGATGA	CTTGGAGAGAAGAACCCT
ETEC	*STh_STp*	GCTAAACCAGYAGRGTCTTCAAAA	CCCGGTACARGCAGGATTACAACA	TGGTCCTGAAAGCATGAA
		TGAATCACTTGACTCTTCAAAA	GGCAGGATTACAACAAAGTT	TGAACAACACATTTTACTGCT
STEC	*stx1/Stx2*	ACTTCTCGACTGCAAAGACGTATG	ACAAATTATCCCCTGWGCCACTATC	CTCTGCAATAGGTACTCCA
		CCACATCGGTGTCTGTTATTAACC	GGTCAAAACGCGCCTGATAG	TTGCTGTGGATATACGAGG
Helicobacter pylori	*ureC*	GACACCAGAAAAAGCGGCTA	AGCGCATGTCTTCGGTTAAA	TCACTAAAGCGTTTTCTACC
Giardia spp	*18s rRNA*	GACGGCTCAGGACAACGGTT	TTGCCAGCGGTGTCCG	CCCGCGGCGGTCCCTGCTAG

Campylobacter Jejuni /coli	*cadF*	CTGCTAAACCATAGAAATAAAATTTCTCAC	CTTTGAAGGTAATTTAGATATGGATAATCG	CATTTTGACGATTTTTGGCTTGA
L. monocytogens	*hly*	TTTCATCCATGGCACCAC	ATCCGCGTGTTTCTTTTCG	CGCCTGCAAGTCCTAAGACGCCA
Clostridium difficile	*tcdB*	GGTATTACCTAATGCTCCAAATAG	TTTGTGCCATCATTTTCTAAGC	CCTGGTGTCCATCCTGTTTC
Salmonella enterica	*ttr*	CTCACCAGGAGATTACAACATGG	AGCTCAGACCAAAAGTGACCATC	CACCGACGGCGAGACCGACTTT
MS2	*MS2g1*	TGGCACTACCCCTCTCCGTATTCAC	GTACGGGCGACCCCACGATGAC	CACATCGATAGATCAAGGTGCCTACAAGC

Table S1: Primers and probes used to test enteric pathogens in soil samples.
