# Supplementary material for: Spatial–Temporal Patterns in the Enteric Pathogen Contamination of Soil in the Public Environments of Low- and Middle-Income Neighborhoods in Nairobi, Kenya
Source: Int J Environ Res Public Health. 2024 Oct 12;21(10):1351. doi: 10.3390/ijerph21101351 (PMC11506941; doi:10.3390/ijerph21101351)
Supplement: Supplementary file 1 [file ijerph-21-01351-s001.zip › Supplementary file 2.pdf]

Table S2: The limit of detection and the average Ct-value of the enteric pathogens for the positive samples

No	Pathogens	Gene(s)	Limit of detection
1	*Shigella* backbone	*ipaH*	32
2	*Shigella* plasmid	*virG*	34
3	*Salmonella enterica*	*ttr*	34
4	*Listeria monocytogens*	*hly*	34
5	*Campylobacter jejuni /coli*	cadF	35
6	*Enteroaggregative E. coli*	aaic/aatA	34
7	*Enteropathogenic E. coli*	eae	33
8	*Enteropathogenic E. coli*	bfpa	33
9	*Enterotoxigenic E. coli*	*sth_stp*	34
10	*Shiga toxin-producing E. coli*	stx1/Stx2	34
11	*Enterotoxigenic E. coli*	*LT*	32
12	Enterovirus	*5'UTR*	34
13	*Clostridium Difficile*	*tcdB*	33
14	*Enterocytozoon Bieneusi*	*ITS*	34
15	*Entamoeba histolytica*	*18S rRNA*	35
16	Norovirus GI	*ORF 1-2*	35
17	Norovirus GII	*ORF 1-2*	33
18	*Cryptosporidium* SPP	*NSP3*	34
19	Sapovirus	*RdRp*	34
20	*Giardia* spp.	*18s rRNA*	32
21	Adenovirus 40/41	*Fiber Gene*	35
22	*Helicobacter Pylori*	*ureC*	34
23	Rotavirus	*NSP3*	35
