# Supplementary material for: Spatial–Temporal Patterns in the Enteric Pathogen Contamination of Soil in the Public Environments of Low- and Middle-Income Neighborhoods in Nairobi, Kenya
Source: Int J Environ Res Public Health. 2024 Oct 12;21(10):1351. doi: 10.3390/ijerph21101351 (PMC11506941; doi:10.3390/ijerph21101351)
Supplement: Supplementary file 1 [file ijerph-21-01351-s001.zip › Supplementary file 3.pdf]

Table S3: Spatial distribution and diversity of enteric pathogens in public domain sites soil in Kibera and Jericho, Kenya (N=160).

Neighborhood	site	Pathogens	Diversity	Total
**Jericho**	Site 1	EAEC (6), STEC (5), ETEC (6), EPEC (5), *Salmonella (2)*	5	24
	Site 2	*EAEC (3), ETEC (1), EPEC (3), E. bieneusi (1)*	4	8
	Site 3	*Campylobacter* (4), ETEC (2), *STEC (1)*	3	7
	Site 4	EAEC (5), EPEC (3), ETEC (3), *Campylobacter (4),* STEC (1)	5	16
	Site 5	EAEC (3), EPEC (4), ETEC (3), STEC (3), *Giardia* (1), Norovirus (1)	6	15
	Site 6	EAEC (4), EPEC (5), ETEC (2), STEC (5)	4	16
	Site 7	EAEC (8), EPEC (7), ETEC (8), STEC (6), *H. pylori (1), E. histolytica (1),* Rotavirus *(1), Salmonella (1)*	8	33
	Site 8	EAEC (3), STEC (1), ETEC (2), EPEC (2), *E. bieneusi* (1)	5	9
	Site 9	EAEC (6), EPEC (4), ETEC (5), STEC (2)	4	17
	Site 10	EAEC (), EPEC (1), ETEC (1)	3	3
	**Average**		**4.7**	14.8
**Kibera**	Site 1	*E. histolytica (1),* EAEC (7), Enterovirus *(1),* EPEC (8), ETEC (8), STEC (7)	6	32
	Site 2	EAEC (7), EPEC (4), ETEC (4), STEC (2), *Shigella (1)*	5	18
	Site 3	EAEC (7), EPEC (6), ETEC (7), STEC (6)	4	26
	Site 4	EAEC (6), EPEC (4), ETEC (5), STEC (2) *Campylobacter (6),* Enterovirus *(1)*	6	24
	Site 5	EAEC (7), EPEC (7), ETEC (7), STEC (2), *Shigella (6), Salmonella (1)*	6	30
	Site 6	EAEC (7), EPEC (7), ETEC (7), STEC (2)	4	23
	Site 7	EAEC (7), EPEC (7), ETEC (7), STEC (2), Adenovirus *(1), Salmonella (1)*	6	25
	Site 8	*EAEC (7), EPEC (4), ETEC (6)*	3	17
	Site 9	EAEC (8), EPEC (7), ETEC (7), STEC (3), *Shigella* (1)	5	26
	Site 10	EAEC (6), EPEC (4), ETEC (2), STEC (1)	4	13
	**Average**		**5**	**23.4**

EAEC = Enteroaggregative E. coli; ETEC = Enterotoxigenic E. coli; EPEC = Enteropathogenic E. coli; STEC = Shiga toxin-producing E. coli. Numbers in the parenthesis indicates the number of each pathogen detected from eight samples in each respective sampling sites.
